# Meta-analysis of sequence-based association studies across three cattle breeds reveals 25 QTL for fat and protein percentages in milk at nucleotide resolution

**DOI:** 10.1186/s12864-017-4263-8

**Published:** 2017-11-09

**Authors:** Hubert Pausch, Reiner Emmerling, Birgit Gredler-Grandl, Ruedi Fries, Hans D. Daetwyler, Michael E. Goddard

**Affiliations:** 1 Animal Genomics, Institute of Agricultural Sciences, ETH Zurich, 8092 Zurich, Switzerland; 2 Agriculture Research Division, Agriculture Victoria, Department of Economic Development, Jobs, Transport and Resources, AgriBio, VIC 3083 Australia; 3 Institute of Animal Breeding, Bavarian State Research Center for Agriculture, 85586 Grub, Germany; 4 QualitasAG, 6300 Zug, Switzerland; 5 Animal Breeding, Technische Universitaet Muenchen, 85354 Freising, Germany; 6 School of Applied Systems Biology, LaTrobe University, Bundoora, VIC 3083 Australia; 7 Faculty of Veterinary and Agricultural Sciences, University of Melbourne, Melbourne, VIC 3010 Australia

**Keywords:** Cattle, Meta-analysis, Sequence imputation, Dairy traits, Qtl

## Abstract

**Background:**

Genotyping and whole-genome sequencing data have been generated for hundreds of thousands of cattle. International consortia used these data to compile imputation reference panels that facilitate the imputation of sequence variant genotypes for animals that have been genotyped using dense microarrays. Association studies with imputed sequence variant genotypes allow for the characterization of quantitative trait loci (QTL) at nucleotide resolution particularly when individuals from several breeds are included in the mapping populations.

**Results:**

We imputed genotypes for 28 million sequence variants in 17,229 cattle of the Braunvieh, Fleckvieh and Holstein breeds in order to compile large mapping populations that provide high power to identify QTL for milk production traits. Association tests between imputed sequence variant genotypes and fat and protein percentages in milk uncovered between six and thirteen QTL (*P* < 1e-8) per breed. Eight of the detected QTL were significant in more than one breed. We combined the results across breeds using meta-analysis and identified a total of 25 QTL including six that were not significant in the within-breed association studies. Two missense mutations in the *ABCG2* (p.Y581S, rs43702337, *P* = 4.3e-34) and *GHR* (p.F279Y, rs385640152, *P* = 1.6e-74) genes were the top variants at QTL on chromosomes 6 and 20. Another known causal missense mutation in the *DGAT1* gene (p.A232K, rs109326954, *P* = 8.4e-1436) was the second top variant at a QTL on chromosome 14 but its allelic substitution effects were inconsistent across breeds. It turned out that the conflicting allelic substitution effects resulted from flaws in the imputed genotypes due to the use of a multi-breed reference population for genotype imputation.

**Conclusions:**

Many QTL for milk production traits segregate across breeds and across-breed meta-analysis has greater power to detect such QTL than within-breed association testing. Association testing between imputed sequence variant genotypes and phenotypes of interest facilitates identifying causal mutations provided the accuracy of imputation is high. However, true causal mutations may remain undetected when the imputed sequence variant genotypes contain flaws. It is highly recommended to validate the effect of known causal variants in order to assess the ability to detect true causal mutations in association studies with imputed sequence variants.

**Electronic supplementary material:**

The online version of this article (10.1186/s12864-017-4263-8) contains supplementary material, which is available to authorized users.

## Background

Whole-genome sequencing data have been generated for a large number of cattle from diverse breeds. Many of the sequenced cattle were selected in a way that they account for a large proportion of the genetic diversity of the entire population in order to ensure that the information content of the sequencing data is high [1]. This so-called “key-ancestor”-approach reveals most polymorphic sites that segregate within breeds, at least the not too rare ones [2]. The availability of a comprehensive catalogue of sequence variants that segregate within and across breeds proved to be useful to pinpoint deleterious mutations particularly for monogenic traits [3, 4].

International consortia such as the 1000 bull genomes project (http://www.1000bullgenomes.com/) collected sequencing data from hundreds to thousands of individuals in order to characterize sequence variation that segregates within and across populations [5]. The fifth run of the 1000 bull genomes project provided genotypes at 39 million polymorphic sites for 1577 individuals that represent the most important dairy and beef breeds in the world. Reference panels that include sequence information from many breeds allow us to impute sequence variant genotypes at high accuracy for animals that have been genotyped using dense microarrays [6, 7]. Association studies with imputed sequence variant genotypes may facilitate to pinpoint causal mutations for complex traits [7, 8].

Although sequence-based association studies uncovered many QTL (e.g., [9–11]), our knowledge regarding their molecular-genetic underpinnings is still limited because the characterization of putatively causal variants was rarely attempted (e.g., [5, 12, 13]). Sequence-based association studies typically reveal nearly identical *P* values for many adjacent variants that are in high linkage disequilibrium (LD). Such a pattern prevents differentiation between true causal mutations and anonymous markers that are in LD with them. Because many QTL for complex traits reside in non-coding regions of the genome, a functional prioritization of significantly associated sequence variants is not always possible [14].

Association studies that include animals from different breeds may improve the resolution of QTL mapping because LD is conserved only over short distances across breeds [15]. However, trait definitions and data recording methods need to be standardized across breeds in order to allow for multi-breed association testing [16]. When restricted access to individual-level data precludes multi-breed association testing, meta-analysis enables us to combine summary statistics of association studies across populations thereby providing high power to detect QTL [17].

In this paper, we report on association studies between imputed sequence variant genotypes and two dairy traits in 17,229 cattle from three breeds. Meta-analysis of association studies across breeds allowed us to characterize 25 QTL for fat and protein percentages in milk at nucleotide resolution.

## Methods

### Genotyped animals of the target populations

All genotype data were obtained from breeding organizations and no new samples were genotyped in this study. The target populations consisted of 1646 Braunvieh (BV), 6778 Fleckvieh (FV) and 8805 Holstein (HOL) bulls that had (partially imputed) genotypes at 573,650, 603,662 and 564,374 autosomal single nucleotide polymorphisms (SNPs). A subset of the animals (214 BV, 1475 FV and 345 HOL) was genotyped using the Illumina BovineHD (HD) bead chip that comprises 777,962 SNPs. All other animals were genotyped using the Illumina BovineSNP50 Bead chip (50 K) that comprises 54,001 (version 1) or 54,609 (version 2) SNPs. The 50 K genotypes were imputed to higher density using a combination of *Beagle* [18] and *Minimac* [19] (HOL, FV) or *FImpute* [20] (BV) as described previously [3, 21]. To improve the accuracy of imputation, we increased the size of the reference panel by including HD genotypes of another 2070 FV and 842 BV cattle that were available from previous projects. However, these 2912 cattle were not considered for association analyses.

### Sequenced reference animals

Two already existing reference panels were used to impute sequence variant genotypes for the animals of the target populations. Sequence variant genotypes for 8805 HOL cattle were imputed using a multi-breed reference population that consisted of 1147 cattle including 59 BV, 213 FV and 312 HOL cattle that were available from the fourth run of the of the 1000 bull genomes project [5]. The sequencing reads were aligned to the UMD3.1 bovine reference genome using the *BWA*-*MEM* algorithm [22, 23]. Single nucleotide polymorphisms, short insertions and deletions were genotyped for all sequenced animals simultaneously using a multi-sample variant calling approach that was implemented with the *mpileup* module of *SAMtools* [24] and that is described in Daetwyler et al. [5]. The sequence variant genotypes of 1147 reference animals were filtered to include 29,460,467 autosomal sequence variants with a minor allele frequency (MAF) greater than 0.0013 (i.e., the minor allele was observed at least four times).

Sequence variant genotypes for 1646 BV and 6778 FV cattle with (partially imputed) HD genotypes were imputed using a multi-breed reference population that consisted of 1577 animals that were included in the fifth run of the 1000 bull genomes project. The raw sequencing data were processed as described above. The sequence variant genotypes of 1577 reference animals were filtered to include 28,542,148 autosomal sequence variants that segregated in 123, 279 and 451 sequenced animals of the BV, FV and HOL breed, respectively.

A total of 24,180,002 sequence variants were a common subset of both reference panels. Sequence variant genotypes were imputed separately for each breed using a pre-phasing-based imputation approach that was implemented in the *Minimac* [25] software tool. Haplotype phases for the reference animals were estimated using *Beagle* (version 3.2.1) [18] (HOL) or *Eagle* (version 2.3) [26] (FV, BV). Sequence variants that were located between 71 and 78 Mb on chromosome 12 or between 23 and 30 Mb on chromosome 23 were not considered for association testing because the accuracy of imputation was very low within both segments [7].

### Within-breed association testing

Association tests between imputed sequence variants and fat (FP) and protein percentages (PP) in milk were carried out for each breed separately using a variance components-based approach that was implemented in the *EMMAX* software tool and that accounts for population stratification and relatedness by fitting a genomic relationship matrix [27]. A genomic relationship matrix was built for each breed based on (partially imputed) HD genotypes using the method of Yang et al. [28] that was implemented in the *plink* (version 1.9) software tool [29]. Daughter yield deviations (DYDs) for FP and PP with an average reliability of 0.92 (±0.04) were the response variables in FV. Estimated breeding values (EBVs) with an average reliability of 0.89 (±0.12) and 0.95 (±0.03) were the response variables in BV and HOL. The phenotypic correlation between FP and PP was 0.53, 0.64 and 0.69 in BV, FV and HOL cattle. Predicted allele dosages were used as explanatory variables for the association tests. Sequence variants with *P* values less than 1e-8 were considered as significantly associated.

### Identification of QTL that segregate within and across breeds

Genomic regions with significantly associated variants were inspected manually. Genes that were annotated within 1 Mb intervals centered on the top variant were extracted from the UMD3.1 annotation of the bovine genome [30] using the Reference Sequence database (RefSeq release 82) from the National Center for Biotechnology Information (NCBI) and compared to known QTL for bovine milk production traits using literature review. Genomic regions were considered as across-breed QTL when significantly associated sequence variants (*P* < 1e-8) were located within a 1 Mb interval centered on top variants that were detected at P < 1e-8 in another breed.

### Meta-analysis of FP and PP across three breeds

Estimated allelic substitution effects and corresponding standard errors from the within-breed association studies (see above) were divided by phenotypic standard deviations in order to standardize the results of the association studies across breeds. Variants with an effect size greater than five standard deviations were not included in the meta-analysis (most of these variants also had low MAF and the large effect possibly results from erroneously imputed alleles). The number of variants that were excluded because they had an effect size greater than five standard deviations varied across breeds and traits and it ranged from 13,461 to 22,079. Meta-analysis was performed using an inverse variance-based approach that takes into account sample size, allelic substitution effect and standard error [17]. Heterogeneity of the effect sizes across breeds was evaluated using Cochran’s Q test [31] that was also implemented in the *METAL* software package [17]. The functional consequence of significantly associated sequence variants was predicted using the *Variant Effect Predictor* tool from Ensembl [32]. Variant-specific estimates of F_ST_ were calculated for 25 QTL and whole genome sequence variants using ${F}_{ST}=\frac{s^2}{\overline{p}\left(1-\overline{p}\right)+{s}^2/r}$, where s^2^ is the sample variance of allele frequency between breeds, $\overline{p}$ is the mean allele frequency across breeds and r is the number of breeds [33, 34].

### Validation of 25 QTL in another population

A validation population that consisted of 1839 FV cows was genotyped at 777,962 SNPs using the HD bead chip. Haplotype phases were estimated using *Beagle* [26]. Sequence variant genotypes were imputed using *Minimac* [19] considering 1147 animals from the fourth run of the 1000 bull genomes project as a reference population (see above). EBVs for FP and PP with an average reliability of 0.50 (±0.03) were used as response variables for the association tests using the *EMMAX* [27] software tool as described above. Variants that had *P* values less than 0.05 and allelic substitution effects that were in the same direction as in the meta-analysis were considered to be validated in the cow population.

## Results

Genotypes for more than 28 million sequence variants were imputed for 17,229 progeny-tested bulls of the BV, FV and HOL cattle breeds using a population-based genotype imputation approach. Following genotype imputation, we considered 18,063,587, 19,021,606 and 17,318,499 imputed sequence variants that had MAF greater than 0.005 in BV, FV and HOL, respectively, for association testing. Between 32.4 and 35.1% of the imputed sequence variants had MAF less than 0.05 (see Additional file 1: Figure S1). The estimated mean (and median) accuracy of imputation (r^2^-values from *Minimac*) was 0.78 (0.96), 0.80 (0.97) and 0.79 (0.99) in BV, FV and HOL, respectively, and 85.4, 86.7 and 83.6% of the variants were imputed at an estimated accuracy greater than 0.3.

### Within-breed association studies for fat and protein percentages in milk

Association tests between imputed sequence variants and FP and PP were carried out separately for each breed. The number of associated sequence variants was higher in HOL and FV than BV which was likely because of a greater sample size in HOL and FV (BV: 1646, FV: 6778, HOL: 8805); 3249, 11,939 and 15,857 sequence variants were associated (*P* < 1e-8) with FP in BV, FV and HOL, respectively, and 2296, 6515 and 15,674 were associated with PP. The difference in the number of significant variants per trait is mostly attributable to the properties of individual QTL regions. This is exemplified by the *DGAT1* QTL on BTA14, which has a more pronounced effect on FP than PP. In FV and HOL, respectively, 10,046 and 13,401 variants at the proximal region (<10 Mb) of chromosome 14 were associated (P < 1e-8) with FP whereas only 2799 and 9493 variants were associated with PP. Two, 3106 and 10,029 variants were significant for both traits and 5543, 15,348 and 21,502 were significant for at least one trait in BV, FV and HOL, respectively.

**Fig. 1:**
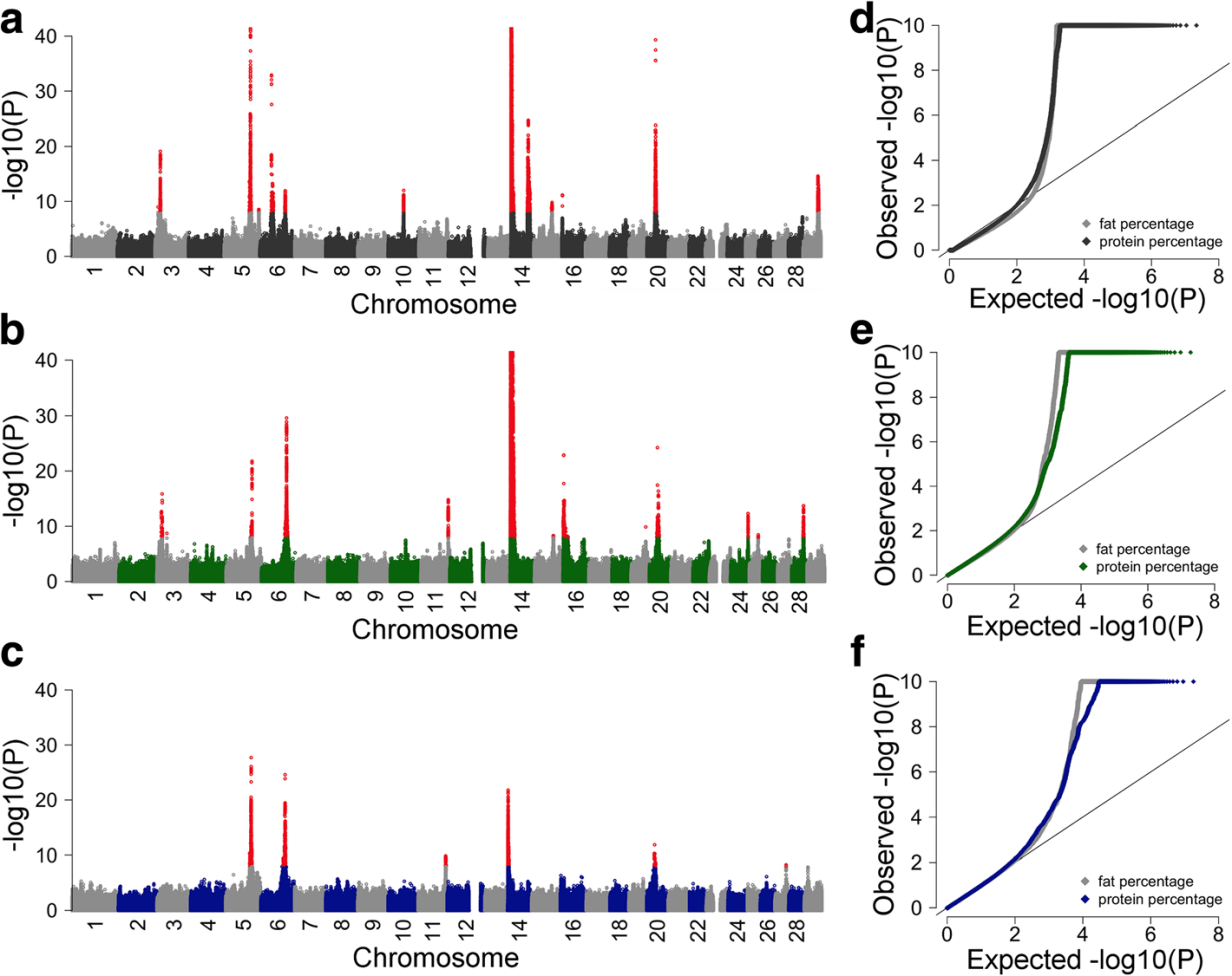
Detection of QTL for dairy traits in three cattle breeds. (**a**-**f**) Composite manhattan and corresponding quantile-quantile (QQ) plots showing the association of 17,318,499, 19,021,606 and 18,063,587 imputed sequence variants, respectively, with FP and PP in HOL (**a**, **d**), FV (**b**, **e**) and BV (**c**, **f**). The composite manhattan plots summarize the results for FP and PP, i.e., each dot shows the more significant *P* value that was observed across both traits. Red colours represent sequence variants with *P* values less than 1e-8. The y-axis of the manhattan and QQ plots is truncated at –log10(1e-40) and –log10(1e-10), respectively

Six, thirteen and twelve QTL, respectively, were detected in BV, FV and HOL cattle (Fig. 1). Eleven QTL were detected in one breed, five and two QTL were detected in two and three breeds, respectively (see Additional file 2: Table S1). However, the top variants at QTL that were significant in more than one breed differed for all but one QTL; rs385640152 was the top variant for a QTL on chromosome 20 in all three breeds analysed.

Although more than 32% of the sequence variants had MAF between 0.005 and 0.05 (see above), only six (19%) QTL had MAF less than 0.05 (see Additional file 2: Table S1). QTL with MAF less than 0.05 were not detected in BV likely because the number of genotyped animals was too low to detect low-frequency QTL.

435 and 201 sequence variants, respectively, were significant (*P* < 1e-8) for FP and PP in all three breeds analysed. Nineteen variants that were associated with FP in all breeds were located on chromosome 5 within the 5′-upstream sequence or intronic regions of *MGST1* (*microsomal glutathione S-transferase 1*). Another 415 variants that were significant for FP in all three breeds were located on chromosome 14 between 1,322,209 and 3,382,844 bp. In FV and HOL, several variants with *P* values less than 5e-306 (i.e., the smallest possible P value that can be obtained using the *EMMAX* software tool) including the p.A232K-variant (rs109326954) in the *DGAT1* (*diacylglycerol O-acyltransferase 1*) gene were located within this segment. When variants with P values less than 5e-306 were ranked according to their t values (i.e., regression coefficient divided by its standard error), rs109326954 was the second top variant in FV and its t-value (53.06) was only slightly less than the top variant (*t* = 53.08, rs209876151 at 1,800,439 bp). In HOL, the t-value of rs109326954 was 2.7 points less than the top variant (rs110568020 at 1,699,681 bp). rs109326954 was not significant for FP in BV. At a QTL on chromosome 20, the p.F279Y-variant (rs385640152 at 31,909,478 bp) in the *GHR* (*growth hormone receptor*) gene was the top variant in all three breeds analysed.

221 non-coding sequence variants that were located between 87,154,594 and 87,434,710 bp on chromosome 6 and rs385640152 in the *GHR* gene were significant for PP in all breeds analysed.

**Fig. 2:**
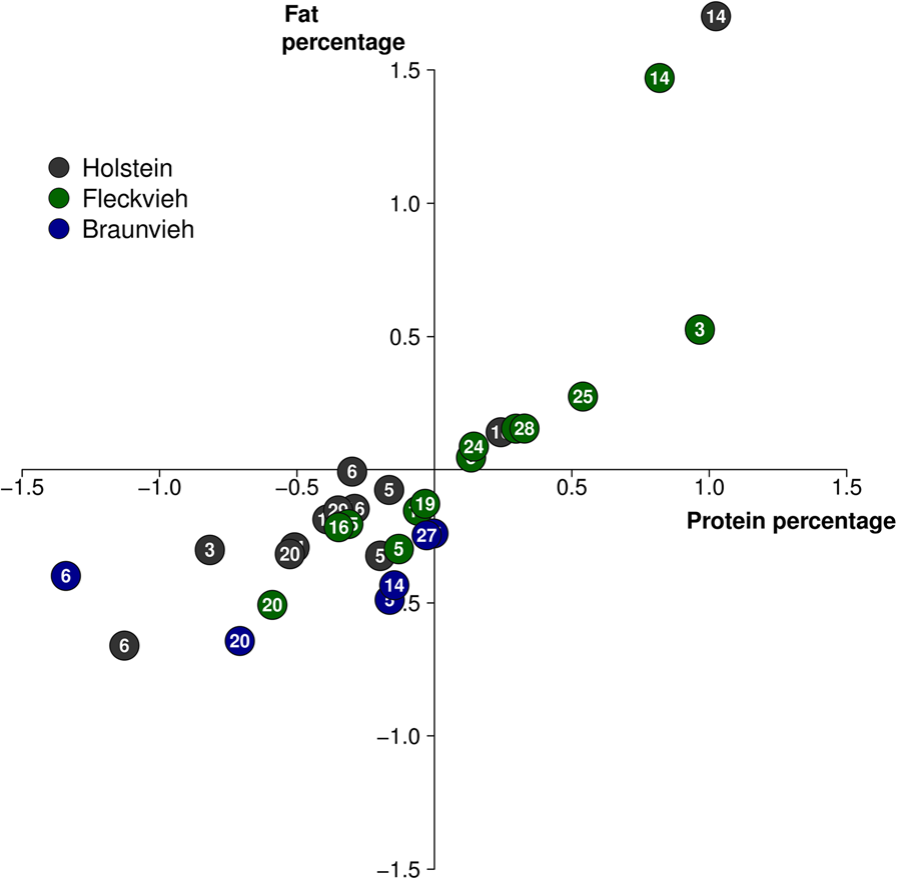
Effect of 31 QTL on fat and protein percentages in milk. Allelic substitution effects of six, thirteen and twelve QTL, respectively, that were detected in HOL (grey), FV (green) and BV (blue) cattle. The allelic substitution effects of the top variants were divided by phenotypic standard deviations to standardize QTL effects across breeds

Considering the allelic substitution effects of 18,063,587, 19,021,606 and 17,318,499 sequence variants in BV, FV and HOL, respectively, the correlation between FP and PP was 0.52, 0.61 and 0.53. The correlation between the FP and PP allelic substitution effects of 19 detected QTL was 0.81 (Fig. 2). The largest effects were detected for three QTL on chromosomes 14, 20 and 6 that encompassed the *DGAT1*, *GHR*, *ABCG2* (*ATP binding cassette subfamily G member 2*) and *CSN1S1* (*casein alpha s1*) genes. The QTL on BTA14 encompassing the *DGAT1* gene had larger effects on FP than PP whereas the effects of the QTL on BTA6 and BTA20 were more pronounced for PP than FP.

### Multi-breed meta-analysis uncovers six additional QTL

**Fig. 3:**
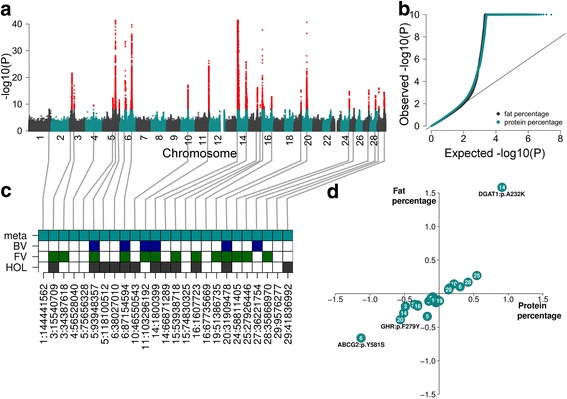
Meta-analysis of fat and protein percentages in milk across three cattle breeds. (**a**) Composite manhattan plot that shows the association of 26,473,121 imputed sequence variants with FP and PP in the meta-analysis. The composite manhattan plot summarizes the results of the meta-analyses, i.e., each dot shows the more significant P value that was observed across both traits. Red colours represent sequence variants with P values less than 1e-8. The y-axis is truncated at –log10(1e-40). (**b**) Quantile-quantile plot of the meta-analyses. Grey and cyan colour represent P values of 26,473,121 imputed sequence variants for FP and PP, respectively. The y-axis is truncated at –log10(1e-10). (**c**) Overview of 25 QTL that were significant at *P* < 1e-8 in the meta-analysis and within-breed association studies. Filled squares indicate that QTL were significant in the respective analysis. The labels at the x-axis represent the positon of the top variant at each QTL. (**d**) Allelic substitution effects of 25 OTL on FP and PP. The QTL effects are given in phenotypic standard deviations. Bold type indicates three causal missense mutations in the *ABCG2*, *DGAT1* and *GHR* genes

Meta-analysis of the within-breed association studies across three breeds revealed 16,086 and 14,020 sequence variants that were significantly associated (*P* < 1e-8) with FP and PP, respectively (see Additional file 3: Table S2 & Additional file 4: Table S3). 23,786 variants were significant for at least one trait and 6320 variants were significant for both traits. The significant sequence variants clustered at 25 QTL including six that did not meet the significance threshold (P < 1e-8) in any of the within-breed association studies (Table 1, Fig. 3).

**Table 1:** Top variants at 25 QTL for fat and protein percentages in milk

QTL	Top variant					Fat percentage			Protein percentage		
	Chr	Position	NCBI rs-ID	Alternate allele	Candidate gene(s)	Allele substitution effect (±standard error)	Effect direction (and evidence of heterogeneity) across three populations^a^	*P*_meta_	Allele substitution effect (±standard error)	Effect direction (and evidence of heterogeneity) across three populations^a^	*P*_meta_
1^c^	1	144,441,562	rs136426342	G	*SLC37A1*	−0.05 (±0.015)	--- (0.44)	1.2e-03	−0.09 (±0.016)	--- (0.37)	8.5e-09
2	3	15,540,709	rs207616487	C		−0.19 (±0.038)	?-- (0.11)	5.6e-07	−0.49 (±0.05)	--- (0.26)	3.0e-22
3	3	34,387,618	rs109030498	T		0.06 (±0.014)	+++ (0.18)	9.1e-05	0.14 (±0.016)	+++ (0.88)	1.1e-17
4^c^	4	56,528,040	rs381550282	A		0.08 (±0.022)	+++ (0.61)	2.0e-04	0.15 (±0.024)	+++ (0.74)	2.5e-10
5^c^	5	75,656,328	rs210334611	T	*TST*	−0.11 (±0.019)	--- (0.27)	6.1e-09	−0.15 (±0.021)	--- (0.48)	6.2e-13
6	5	93,948,357	rs209372883	C	*MGST1*	−0.34 (±0.016)	--- (1.1e-3)	9.8e-100	−0.17 (±0.018)	--- (0.23)	6.3e-21
7	5	118,100,512	rs384440535	T	*TBC1D22A*	−0.07 (±0.015)	+ − - (0.09)	1.1e-05	−0.12 (±0.017)	--- (0.28)	3.0e-12
8	6	38,027,010	rs43702337	C	*ABCG2*	−0.66 (±0.075)	??- (1)	1.1e-18	−1.13 (±0.093)	??- (1)	4.3e-34
9	6	87,154,594	rs109193501	G	*CSN1S1*	0.10 (±0.016)	+++ (1.5e-4)	4.1e-10	0.30 (±0.018)	+++ (9.1e-3)	4.9e-68
10	10	46,550,543	rs208077205	A		0.15 (±0.023)	+++ (0.68)	3.7e-10	0.22 (±0.025)	+++ (0.51)	8.2e-18
11	11	103,296,192	rs381989107	C	*PAEP*	−0.14 (±0.013)	--- (1.0e-3)	6.1e-27	−0.05 (±0.014)	--- (0.43)	6.8e-04
12^b^	14	1,802,266	rs109326954	A	*DGAT1*	1.58 (±0.02)	−++ (1.9e-12)	8.4e-1436	0.91 (±0.024)	−++ (2.3e-5)	1.9e-308
		1,800,399	rs208317364	A		1.59 (±0.02)	−++ (2.8e-14)	1.2e-1437	0.91 (±0.024)	−++ (6.2e-6)	1.1e-308
13	14	66,871,289	rs439256148	C		−0.29 (±0.037)	+ − - (0.25)	4.4e-15	−0.51 (±0.048)	--- (0.50)	1.6e-26
14	15	53,938,718	rs386031410	A		−0.17 (±0.034)	+ − - (0.25)	8.1e-07	−0.31 (±0.037)	+ − - (0.43)	6.3e-17
15^c^	15	74,830,325	rs42364320	T		−0.09 (±0.014)	--- (0.92)	2.1e-10	−0.02 (±0.016)	+ − - (0.68)	0.13
16	16	1,607,723	rs382661583	T		−0.19 (±0.022)	--- (0.33)	1.3e-17	−0.31 (±0.024)	--- (0.27)	1.6e-38
17^c^	16	67,735,669	rs385934483	A		−0.1 (±0.013)	--- (0.28)	1.9e-13	−0.07 (±0.015)	--- (0.09)	5.8e-06
18	19	51,386,735	rs137372738	T	*FASN*	−0.11 (±0.013)	--- (0.22)	5.9e-16	0.01 (±0.014)	+++ (0.96)	0.72
19	20	31,909,478	rs385640152	T	*GHR*	−0.39 (±0.028)	--- (4.1e-4)	2.9e-45	−0.56 (±0.031)	--- (0.19)	1.6e-74
20	24	58,811,405	rs380879212	A	*LMAN1*	0.07 (±0.016)	+++ (0.59)	4.5e-05	0.14 (±0.017)	+++ (0.91)	1.9e-15
21	25	27,926,446	rs801168123	A		0.27 (±0.09)	?++ (0.44)	2.6e-03	0.55 (±0.093)	?++ (0.45)	4.5e-09
22	27	36,221,754	rs208624037	G	*AGPAT6*	−0.12 (±0.015)	--- (0.002)	6.1e-16	−0.03 (±0.016)	--- (0.39)	0.11
23	28	35,868,970	NA	A	*MBL1*	0.17 (±0.054)	? +? (1)	1.7e-03	0.42 (±0.056)	? +? (P = 1)	7.1e-14
24^c^	29	9,576,277	rs384716744	T		0.06 (±0.015)	+++ (0.07)	6.1e-05	0.14 (±0.016)	+++ (0.01)	1.1e-16
25	29	41,836,992	rs471090482	G		−0.16 (±0.04)	??- (1)	5.8e-05	−0.35 (±0.045)	??- (1)	6.6e-15

^a^ Populations in order are BV, FV and HOL; ‘+’ and ‘–‘denote positive and negative substitution effects of the alternate allele. ‘?’ indicates that the variant did not segregate in the respective population. The P value of Cochran’s Q test for heterogeneity of the effect sizes across breeds is given in parentheses

^b^ The *P* value of the top variant (rs208317364) was only slightly less than for a known causal variant (rs109326954) in the *DGAT1* gene with a major effect on milk composition in cattle. For completeness, the effect of rs109326954 is shown as well

^c^ QTL was not significant at P < 1e-8 in the within-breed analyses

The top variants at most QTL (20/25) had MAF greater than 0.005 in all three breeds analysed. However, the power of the within-breed association studies was likely not sufficient to detect all of them at P < 1e-8 (see Additional file 5: Table S4). Seven QTL showed evidence of across-breed heterogeneity of the allelic substitution effects at *P* < 0.05. The genetic differentiation of the three breeds was greater at 25 QTL (F_ST_ = 0.079) than 24,180,002 genome-wide sequence variants (F_ST_ = 0.059) indicating that at least some QTL were targets of recent selection.

The top variants at 19 QTL had MAF greater than 0.01 in a validation population that consisted of 1839 FV cows. The allelic substitution effects of 16 variants had *P* values less than 0.05 in the validation population and were in the same direction as in the meta-analysis (see Additional file 6: Table S5).

The most significantly associated variant at a QTL on chromosome 6 was a known causal mutation for milk production traits (p.Y581S, rs43702337 at 38,027,010 bp, *P* = 4.3e-34) in the *ABCG2* gene [35]. The serine-encoding C-allele had a frequency of 0.008 in HOL and it decreased FP and PP (Fig. 3d, Table 1). The serine variant did not segregate in the BV and FV bulls that had imputed sequence variant genotypes.

The p.F279Y-variant in the *GHR* gene (rs385640152 at 31,909,478, *P* = 1.6e-74) was the top variant at a QTL on BTA20. Consistent with previous findings, the tyrosine-encoding T-allele decreased FP and PP in all three breeds analysed [8, 36]. The frequency of the tyrosine variant was 0.06, 0.05 and 0.15 in BV, FV and HOL (**see** Additional file 2: Table S1). The p.S18 N variant (rs136247583) in the *PRLR* (*prolactin receptor*) gene [37] was polymorphic in the three breeds analysed and the frequency of the asparagine variant was 0.87 (HOL), 0.25 (FV) and 0.14 (BV). However, rs136247583 was neither associated with FP and PP in the meta-analysis (P_FP_ = 0.82, P_PP_ = 0.06) nor in any of the within-breed association studies (P_FP_ > 0.17, P_PP_ > 0.15).

A QTL on chromosome 1 was associated with FP in the meta-analysis but not in the within-breed association studies. Six imputed sequence variants that were located downstream of the *SLC37A1* (*solute carrier family 37 member 1*) gene had *P* values less than 1e-8. The variants associated with FP were in immediate vicinity to a QTL for phosphorus concentration in milk that was detected in an Australian Holstein cattle population [13]. The top variant (rs136426342 at 144,441,562 bp) of the meta-analysis was less than 75 kb away from two variants in high LD (rs109254133 at 144,367,474 bp and rs208161466 at 144,377,960 bp) that were plausible causal mutations in Holstein cattle [13]. However, their P values (*P* = 3.2e-7 and *P* = 8.0e-7) were higher than the P value (8.6e-9) of the top variant.

The meta-analysis revealed three distinct QTL on chromosome 5; 590 significantly associated sequence variants were located between 75,633,853 and 75,790,113 bp. This interval encompassed the *NCF4* (*neutrophil cytosolic factor 4*) and *CSF2RB* (*colony stimulating factor 2 receptor beta common subunit*) genes, which we did not consider as candidate genes for milk production traits. However, the top variant was less than 20 kb downstream of the translation end of the *TST* (*thiosulfate sulfurtransferase*) gene, which we considered as a functional candidate gene. Another QTL on BTA5 encompassed 1916 significantly associated sequence variants that were located between 88,235,301 and 98,233,638 bp. The effect of his QTL was more pronounced on FP than PP in all three breeds analysed. The most significantly associated variant (rs209372883 at 93,948,357 bp, *P* = 9.8e-100) was located in the second intron of the *MGST1* gene. However, the top variant at that QTL differed across breeds. Another QTL on BTA5 encompassed 36 significantly associated sequence variants that were located between 118,086,877 and 118,264,313 bp including a missense mutation (p.R355C) in the *TBC1D22A* (*TBC1 domain family member 22A*) gene. However, the *P* value of the missense mutation was clearly higher (*P* = 3.2e-9) than the most significantly associated non-coding variant (rs384440535 at 118,100,512 bp, P = 3e-12).

The allelic substitution effects of most QTL were in the same direction for FP and PP (Table 1). However, two QTL on chromosomes 19 and 27 were highly significantly associated with FP (*P* < 5.9e-16) but not with PP (*P* > 0.1). The top variants at these QTL were located in intronic regions of the *FASN* (*fatty acid synthase*) and *AGPAT6* (*1-acylglycerol-3-phosphate O-acyltransferase 6*) genes.

### Conflicting effects of a known causal variant across breeds

The p.A232K-variant (rs109326954) in the *DGAT1* gene was the second top variant in the FP meta-analysis and its P value (8.4e-1436) was marginally higher than the top variant (rs208317364 at 1,800,399 bp, *P* = 1.2e-1437). However, the allelic substitution effect of rs109326954 was not consistent across three breeds analysed. While the lysine variant increased FP and PP in FV and HOL, it decreased both traits in BV. The effect of rs109326954 on FP and PP was significant in FV and HOL but not in BV cattle (*P* > 0.2). The lysine variant had a frequency of 0.08, 0.09 and 0.26 in the BV, FV and HOL animals, respectively, that had imputed genotypes. The allele frequency was similar in the sequenced FV (0.06) and HOL (0.30) animals. However, it was only 0.004 in 123 sequenced BV animals (i.e., only one animal was heterozygous). Moreover, the accuracy of imputation (r^2^-value from *Minimac*: 0.02) was very low for rs109326954 in BV cattle. Taken together, these findings indicate that the imputed genotypes at rs109326954 were flawed in BV which caused the conflicting allelic substitution effects.

## Discussion

Our meta-analysis of association studies for FP and PP across three cattle breeds discovered 25 QTL including six that were not detected at *P* < 1e-8 in the within-breed analyses. Our findings show that including data from several breeds can increase the power of association studies with imputed sequence variant genotypes which agrees with van den Berg et al. [15]. The power to detect QTL may be even greater in multi-breed association studies [38]. However, access to individual-level genotype data was restricted in our study which prevented us from analyzing raw data from all three breeds simultaneously. The imputation reference panels and imputation software differed across the three breeds analyzed, which might affect the power of our meta-analysis. Considering that our study included sequence variant genotypes of more than 17,000 animals, the identified QTL are likely to be the major genetic determinants of milk production traits in BV, FV and HOL cattle. We may have missed QTL with small effects because we applied a rather conservative significance threshold in the meta-analysis. Applying a less stringent threshold and QTL mapping approaches that consider all variants simultaneously may reveal such QTL [39].

We identified a number of QTL for milk production traits that were previously detected in several cattle breeds, e.g., QTL that were located nearby the *SLC37A1*, *MGST1*, *ABCG2*, *CSN1S1*, *PAEP* (*progestagen associated endometrial protein*), *DGAT1*, *FASN*, *GHR* and *AGPAT6* genes [5, 7, 8, 12, 13, 21, 35, 40, 41]. Using imputed sequence variant genotypes, Daetwyler et al. [5] identified a QTL for FP in early lactation in FV and HOL cattle that encompassed the *AGPAT6* gene. Our meta-analysis also identified significantly associated sequence variants (including the variants reported by Daetwyler et al. [5]) in the region 5′-upstream of the *AGPAT6* gene corroborating that this region controls milk fat content in dairy cattle. However, our within-breed analyses did not detect that QTL in HOL and FV cattle at *P* < 1e-8, although the animals of our study were from the same populations and our sample size was two and four times greater compared to Daetwyler et al. [5]. While Daetwyler et al. considered phenotypes for FP in early lactation as response variables, we used phenotypes for FP across the entire lactation. Milk fat content is under different genetic control across the lactation cycle [42] and the findings of Daetwyler et al. [5] and our study indicate that sequence variation nearby *AGPAT6* primarily controls fat content in early lactation which agrees with an expression maximum of *AGPAT6* at an early stage of lactation [43].

Two well characterized missense mutations in the *ABCG2* [35] and *GHR* [36] genes were the top variants at QTL on chromosomes 6 and 20, respectively, demonstrating that causal mutations can be readily identified in association studies with imputed sequence variant genotypes. Moreover, another well-characterized missense mutation in the *DGAT1* gene [44, 45] was the second top variant at a QTL on chromosome 14 and its *P* value was only marginally higher than the top variant. Considering that the meta-analysis revealed three known causal mutations as the top (or second top) variants, it is likely that true causal mutations for milk production traits were among the significantly associated sequence variants detected at other QTL. However, most associated variants resided in non-coding regions of the genome and a functional characterization of such variants was not attempted in our study. Nevertheless, including the trait-associated sequence variants of our meta-analysis in genomic predictions may improve the reliability of genomic breeding values for dairy traits in cattle also for breeds other than BV, FV and HOL [46–48].

A well-characterized causal mutation (p.A232K, rs109326954) in the *DGAT1* gene [44, 45] was the second top variant at a QTL on chromosome 14. In agreement with previous findings [8, 44, 45], the lysine variant was associated with higher FP and PP in FV and HOL. However, it had conflicting effects in BV cattle. A criterion for causality is consistency of allelic substitution effects across breeds. Strictly applying this criterion would lead to the exclusion of the p.A232K-variant as a plausible causal mutation. Closer inspection of the genotypes revealed that the lysine variant had a frequency of 0.004 and 0.08, respectively, in 123 sequenced and 1646 imputed BV animals. According to previous studies, the alanine variant is (nearly) fixed in BV cattle [45, 49], which corroborates that the genotypes of the sequenced reference animals are correct. Since the p.A232K was imputed at very low accuracy (r^2^ = 0.02), it is likely that the conflicting allele frequencies and allelic substitution effects resulted from flaws in the imputed genotypes. Excluding variants with low r^2^-values would have removed the p.A232K variant from the BV population [50]. However, using too stringent cutoff values also carries the risk to exclude from the data well-imputed genotypes [50, 51]. Thus, we decided not to filter the imputed sequence variants based on their r^2^-values. We used reference populations that included animals from diverse populations to impute sequence variant genotypes. Multi-breed reference panels include many sequence variants that are not polymorphic in the target population(s). While multi-breed reference panels may enable us to impute genotypes at high accuracy (e.g., [6, 7, 52]), our findings also show that they may promote the imputation of alleles that do not segregate in the target population. Our findings indicate that it might be advisable to compile breed-specific imputation reference panels that include animals from diverse breeds but only variants that segregate in the sequenced animals from the target breed [7, 53]. Such an approach would likely remove from the data a significant proportion of variants with flaws in the imputed genotypes [53]. However, this approach is only applicable when many individuals of the target breed have already been sequenced in order to determine which sequence variants segregate in the population of interest.

Not all QTL identified in the meta-analysis (Table 1) were significant in all breeds. This could be because a QTL does not segregate in the three breeds analyzed or because we lacked power to show that it was significant at *P* < 1e-8 particularly if it had a low frequency [54]. At five QTL, the top variant was not polymorphic in all breeds and so most likely the QTL does not segregate in those breeds. In addition, *DGAT1* had one very rare allele in BV which was likely to be imputed erroneously. Apart from these six QTL, there is only one case where the QTL does not have an effect in the same direction in all breeds for the more significant trait. This suggests that most detected QTL segregate in all three breeds even though they were not significant in the within-breed analysis.

The correlation between the effects of these QTL on FP and PP is a little surprising because the pathways for fat and protein synthesis in milk are quite different. One reason for this correlation is a QTL that affects milk volume without an equally large change in fat or protein yield and hence changes both PP and FP in the same direction. *ABCG2* and *GHR* might be examples of this [35, 36]. Some QTL which have a functional role in fat synthesis (*DGAT1*, *AGPAT6*, *FASN*) have a bigger effect on FP than PP but still have an effect on PP except for *FASN*. Conversely, *CSN1S1* and *PAEP* encode major milk protein components yet they affect both traits in our study. Perhaps the correlation between PP and FP is due in part to competition for substrate between lactose synthesis (which drives milk volume) and fat or protein synthesis so that an increase in either fat or protein can cause a decrease in volume as seen in the case of *DGAT1* [44, 45].

## Conclusions

Many QTL for milk production traits segregate across cattle breeds and meta-analysis of association studies across breeds has greater power to detect such QTL than within-breed association analyses. Sequence variants that are associated with dairy traits often reside in non-coding regions of the genome. True causal variants at milk production QTL can be readily identified in association studies with accurately imputed sequence variant genotypes. However, using reference panels that include animals from many breeds to impute sequence variant genotypes for GWAS populations may also promote the imputation of alleles that are actually not polymorphic in the target population. Such flaws in the imputed sequence variant genotypes can cause inconsistency of allelic substitution effects of true causal mutations across breeds thereby complicating the differentiation between true causal mutations and neutral sequence variants that are in LD with them.

## Additional files

Additional file 1: Figure S1. Allele frequency distribution of imputed sequence variants. Blue, green and grey, respectively, represent the proportion of imputed sequence variants in BV, FV and HOL for ten allele frequency classes. (TIFF 318 kb)

Additional file 2: Table S1. Most significantly associated variants at 19 QTL. Chromosomal position (UMD3.1) and allelic substitution effect of the alternate allele of the most significantly associated variant at six, thirteen and twelve QTL, respectively, that were detected in BV, FV and HOL cattle. The allelic substitution effects were divided by phenotypic standard deviations. Coloured background indicates variants that were top variants within a 1 Mb region. Please note that the causal mutation in the *DGAT1* gene (Chr14:1,802,266) is presented for the sake of completeness although it was not the most significantly associated variant in any breed. (XLSX 45 kb)

Additional file 3: Table S2. Sequence variants associated (*P* < 1e-8) with fat percentage**.** Chromosomal positon (UMD3.1) and *P* values of 16,086 sequence variants that were significantly associated with fat percentage in the across-breed meta-analysis. (CSV 330 kb)

Additional file 4: Table S3. Sequence variants associated (P < 1e-8) with protein percentage**.** Chromosomal positon (UMD3.1) and P values of 14,020 sequence variants that were significantly associated with fat percentage in the across-breed meta-analysis. (CSV 289 kb)

Additional file 5: Table S4. Characteristics of 25 QTL in three breeds analysed. Frequency of 25 QTL in the BV, FV and HOL cattle breeds. The F_ST_ value indicates the variant-specific genetic differentiation across breeds. The P value of the top variant is given for the more significant trait. The position refers to the UMD3.1 assembly of the bovine genome. (XLSX 44 kb)

Additional file 6: Table S5. Association of the top variants at 25 QTL with FP and PP in 1839 FV cows. Characteristics of the top variants at 25 QTL in a validation population of 1839 FV cows. Bold type and ‘NA’ indicates P values less than 0.05 and variants that were not polymorphic in the FV cows, respectively. (XLSX 39 kb)

## Abbreviations

BV: Braunvieh
FP: Fat percentage
FV: Fleckvieh
HD: High density
HOL: Holstein
LD: Linkage disequilibrium
MAF: Minor allele frequency
PP: Protein percentage
QTL: Quantitative trait loci
SNP: Single nucleotide polymorphism
