# Supplementary figures and images for: Meta-analysis of sequence-based association studies across three cattle breeds reveals 25 QTL for fat and protein percentages in milk at nucleotide resolution
Source: BMC Genomics. 2017 Nov 9;18:853. doi: 10.1186/s12864-017-4263-8 (PMC5680815; doi:10.1186/s12864-017-4263-8)

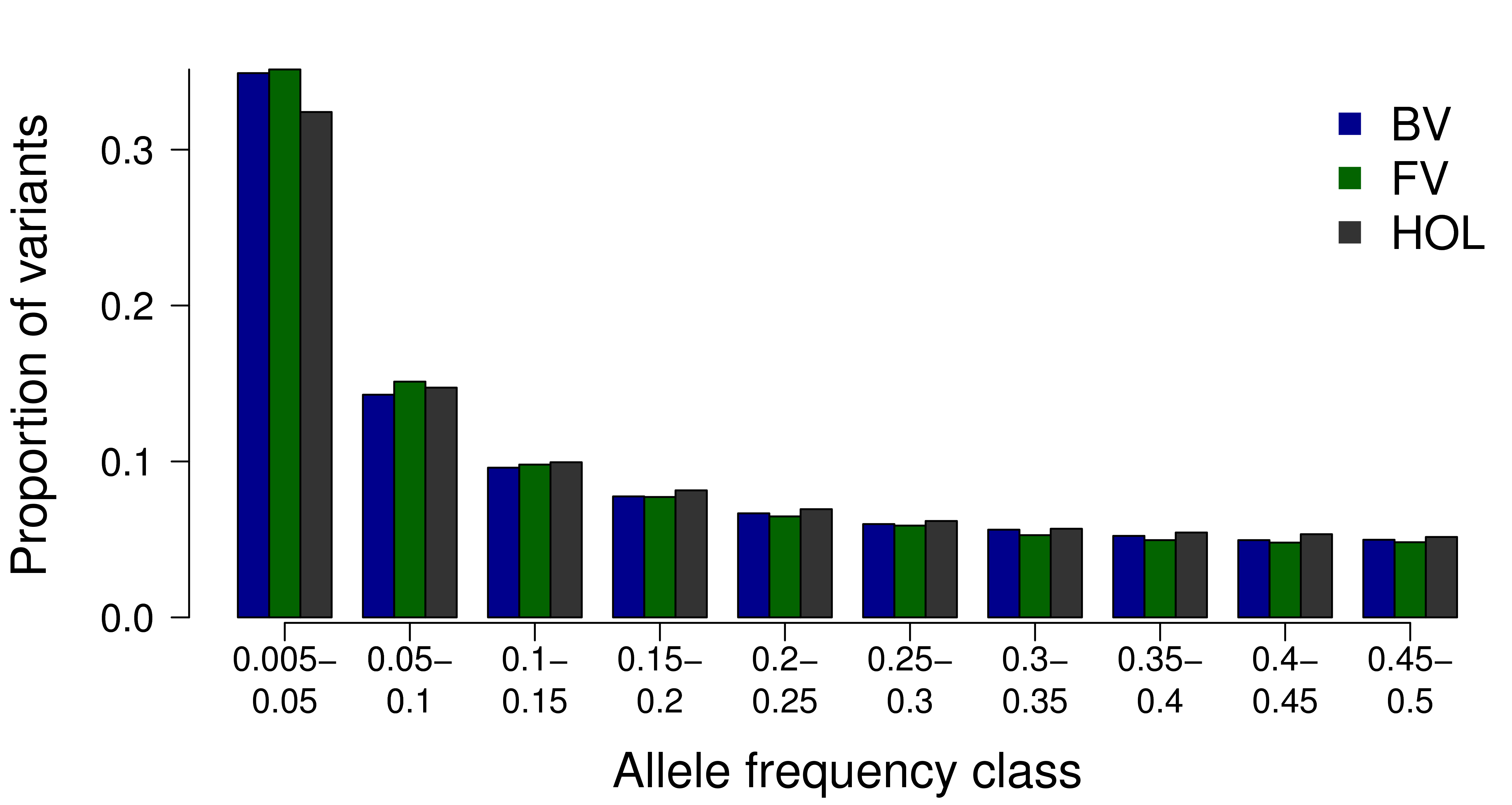

Supplement: Supplementary file 1 — Allele frequency distribution of imputed sequence variants. Blue, green and grey, respectively, represent the proportion of imputed sequence variants in BV, FV and HOL for ten allele frequency classes. (TIFF 318 kb) [file 12864_2017_4263_MOESM1_ESM.tif]
